# Dickkopf-3, a Tissue-Derived Modulator of Local T-Cell Responses

**DOI:** 10.3389/fimmu.2015.00078

**Published:** 2015-02-24

**Authors:** Michael Meister, Maria Papatriantafyllou, Viola Nordström, Varun Kumar, Julia Ludwig, Kathy O. Lui, Ashleigh S. Boyd, Zoran V. Popovic, Thomas Henry Fleming, Gerhard Moldenhauer, Peter P. Nawroth, Hermann-Josef Gröne, Herman Waldmann, Thilo Oelert, Bernd Arnold

**Affiliations:** ^1^Department of Molecular Immunology, German Cancer Research Center, Heidelberg, Germany; ^2^Department of Molecular Pathology, German Cancer Research Center, Heidelberg, Germany; ^3^Department of Medicine I and Clinical Chemistry, University of Heidelberg, Heidelberg, Germany; ^4^Therapeutic Immunology Group, Sir William Dunn School of Pathology, University of Oxford, Oxford, UK; ^5^Department of Pathology, University Medical Center Mannheim, University of Heidelberg, Mannheim, Germany

**Keywords:** Dickkopf-3, T cells, immune privilege, transplantation, autoimmunity

## Abstract

The adaptive immune system protects organisms from harmful environmental insults. In parallel, regulatory mechanisms control immune responses in order to assure preservation of organ integrity. Yet, molecules involved in the control of T-cell responses in peripheral tissues are poorly characterized. Here, we investigated the function of Dickkopf-3 in the modulation of local T-cell reactivity. Dkk3 is a secreted, mainly tissue-derived protein with highest expression in organs considered as immune-privileged such as the eye, embryo, placenta, and brain. While T-cell development and activation status in naïve Dkk3-deficient mice was comparable to littermate controls, we found that Dkk3 contributes to the immunosuppressive microenvironment that protects transplanted, class-I mismatched embryoid bodies from T-cell-mediated rejection. Moreover, genetic deletion or antibody-mediated neutralization of Dkk3 led to an exacerbated experimental autoimmune encephalomyelitis (EAE). This phenotype was accompanied by a change of T-cell polarization displayed by an increase of IFNγ-producing T cells within the central nervous system. In the wild-type situation, Dkk3 expression in the brain was up-regulated during the course of EAE in an IFNγ-dependent manner. In turn, Dkk3 decreased IFNγ activity and served as part of a negative feedback mechanism. Thus, our findings suggest that Dkk3 functions as a tissue-derived modulator of local CD4^+^ and CD8^+^ T-cell responses.

## Introduction

Adaptive immune responses in vital organs have to be precisely coordinated in order to assure proper pathogen clearance and at the same time, to prevent extended immune-mediated inflammation that might impair their function. In particular, this is essential for tissues with limited regenerative capacity such as the central nervous system (CNS), the eye, and the pregnant uterus. In these organs, extensive inflammation is prevented locally by mechanisms such as immune deviation away from destructive immune responses or by active suppression of inflammation.

Besides the self-regulating capacity of the immune system, tissue-resident cells also play an important role in the surveillance of T-cell responses by production of immunosuppressive molecules. For example, programed death-ligand 1 (PD-L1), FasL, and indoleamine 2,3-dioxygenase (IDO) have already been reported to be expressed in peripheral tissues associated with an immunosuppressive micromilieu. PD-L1, a mediator of peripheral T-cell tolerance, is expressed by endothelial cells in the heart, muscle cells, β-cells in the pancreas, and glial cells in the inflamed brain (1–5). Via interaction with its receptor, PD-L1 induces co-inhibitory signals in activated T cells and promotes T-cell apoptosis, anergy, and functional exhaustion (6, 7). Furthermore, FasL, which induces apoptosis of activated T cells, is expressed by neurons, astrocytes, oligodendrocytes, microglia (8), the stroma cells of the retina (9), and on fetal cytotrophoblasts and maternal decidual cells of the placenta (10). Moreover, trophoblasts were shown to produce the enzyme IDO, which catabolizes the amino acid tryptophan, necessary for T-cell survival. The presence of IDO in the trophoblast results in inactivation and apoptosis of T lymphocytes through tryptophan deprivation (11). IDO is also produced by several cell types in the eye (12, 13) and in the brain (14).

*Dickkopf-3* (*Dkk3*) belongs to an evolutionary conserved small gene family, which encodes five secreted glycoproteins, Dkk1-4, and Dkkl1 that share two conserved cysteine-rich domains (15). While Dkk1, 2, and 4 have been shown to induce head formation during embryogenesis via modulation of the Wnt-pathway, Dkk3 is not involved in head development (15, 16). There is though, increasing evidence that it may also interfere with Wnt signaling (17–19). Expression studies revealed highest Dkk3 levels in tissues such as brain, spinal cord, eye, embryo, ovary, and uterus (15, 17).

In a T-cell receptor (TCR)–MHC class-I double transgenic mouse system, we previously identified Dkk3 as a modulator of CD8 T-cell responses. Under these conditions, the induction of antigen-specific, peripheral CD8 T-cell tolerance was dependent on the presence of Dkk3 (20). However, the immunological function of Dickkopf-3 in the physiological situation of a polyclonal T-cell repertoire still remains unclear. Here, we show that Dkk3 functions as a tissue-derived modulator of T-cell responses that locally controls activation and differentiation of T lymphocytes. Our findings provide strong evidence that Dkk3 contributes to the immunoregulatory properties of the tissue microenvironment and therefore may provide a valuable target for immune intervention in autoimmunity, transplantation, and inflammatory disease.

## Materials and Methods

### Mice

C57BL/6N and IFNγR1^−/−^ were obtained from Jackson Laboratories. *Dkk3*^−/−^ mice were provided by Niehrs (21) and backcrossed on C57BL/6N background for more than 10 times. All mice were kept under specific pathogen-free conditions (SPF) at the animal facility of the German Cancer Research Center and used at the age of 8–14 weeks. Animal care was in concordance with the instructions of the regulating authorities.

### Flow cytometry

Surface-antigen staining was performed according to standard protocols using the following antibodies: CD3-PerCpCy5.5 (BioLegend), CD4-PB (BioLegend), CD8-APC-Cy7 (eBioscience), CD25-PE (BD Bioscience), CD44-PeCy7 (BioLegend), CD62L-APC (eBioscience), anti-TCR-Vβ antibodies (BD Bioscience). For intracellular cytokine staining, 1 × 10^6^ lymphocytes were re-stimulated with 50 μg/ml myelin oligodendrocyte-derived glycoprotein (MOG)_33–55_ peptide in the presence of Golgi Plug (BD Bioscience) for 6 h. The staining was performed with Cytofix/Cytoperm reagents (BD Bioscience) according to the manufacturer’s protocol. The following antibodies were used: IL-17-APC (BD Biosciences), GM-CSF-APC (BD Biosciences), IFNγ-APC (BD Biosciences). Intracellular transcription factor staining was performed using a FoxP3 staining buffer set (eBioscience) and a FoxP3-APC antibody (eBioscience) according to the manufacturer’s protocol. Flow cytometry was performed on a FACSCanto (BD Bioscience) with FACS Diva software (BD Bioscience) and analyzed with FlowJo 9.6 (Treestar).

### Embryoid body generation and transplantation

Embryoid bodies (EBs) were generated for transplantation as described previously (22). In brief, ES cell lines were cultured on mitotically inactivated embryonic fibroblasts. Before differentiation, ES cells were passaged on gelatinized flasks in the presence of leukemia-inhibitory factor to maintain their pluripotency. After two passages in the absence of fibroblasts, single-cell suspensions were plated onto dishes of bacteriological plastic to generate EB ready for transplantation at day 14 of culture. Transplantation of EB was carried out as described previously (22). Briefly, two to four EB were grafted under the kidney capsule, and groups of mice were killed for analysis at specified time points. Acceptance of the grafted tissues was defined as an increase in diameter to >5 mm, vascularization, and the lack of leukocyte infiltrate and tissue damage, revealed by histology.

### Experimental autoimmune encephalomyelitis

Experimental autoimmune encephalomyelitis (EAE) was induced by injecting 200 μg MOG_33–55_ peptide emulsified in 100 μl complete Freund’s adjuvant containing 200 μg *Mycobacterium tuberculosis* (Thomas Geyer) s.c. At days 0 and 2 after immunization, each mouse received 200 ng pertussis toxin i.p. (Merck Biosciences). Clinical symptoms were scored as follows: 0, normal; 1, limp tail or hind limb weakness; 2, limp tail and hind limb weakness; 3, partial hind limp paralysis; 4, complete hind limb paralysis; 5, dead or moribund, killed by investigator.

### Immunohistology

Mice were heart-perfused and CNS tissues were fixed with phosphate-buffered 4% formaldehyde. Three-micrometer paraffin sections were de-paraffinized and rehydrated, before staining. For immunohistochemistry, the TSA-Indirect Kit was used (NEN Life Science Products). For fluorescence microscopy, sections were stained with anti-Dkk3, anti-NeuN (Millipore), or anti-GFAP (Millipore) antibodies. As a standard negative control, anti-Dkk3 was substituted by equal amounts of normal mouse IgG (Santa Cruz Biotechnology). Pictures were generated on a cell observer microscope (Zeiss).

### Isolation of lymphocytes from CNS

Experimental autoimmune encephalomyelitis-diseased Dkk3^−/−^ and wild-type (WT) mice were heart-perfused with PBS. Brain and spinal cord was removed and minced in ice-cold PBS with 7% FCS. Remaining pieces were digested in 2.5 mg/ml collagenase D (Roche) and 1 mg/ml DNAse I (Roche) in PBS for 30 min at 37°C and three times mashed through a 40-μm nylon sieve (Falcon). Finally, lymphocytes were isolated by a Percoll gradient (GE Healthcare).

### qRT-PCR

Total RNA was extracted from skin using a FastPrep tissue homogenizer (ThermoScan) and the RNeasy kit (Qiagen). Purified RNA was reverse transcribed using the SuperScript II kit (Invitrogen). Quantitative real-time PCR was performed on a 7500 RT-PCR System (Applied Biosystems) using Absolute qPCR SYBR Green ROX Mix (Thermo Scientific) with a final primer concentration of 200 nM. Primer sequences: *Actb* primer: 5′-TGACAGGATGCAGAAGGAGATTA-3′/5′-AGCCACCGATCCACACAGA-3′; *Dkk3* primer: 5′-TCCCATTGCCACCTTTGG-3′/5′-CCAGTTCTCCAGCTTCAAGTACAC-3′; *Cxcl9* primer: 5′-CTTCGAGGAACCCTAGTGATAAGG-3′/5′-CCTCGGCTGGTGCTGATG-3′; *Cxcl10* primer: 5′-GACGGTCCGCTGCAACTG-3′/5′-CCCTATGGCCCTCATTCTCA-3′; *Ccl2* primer: 5′-AGCAGGTGTCCCAAAGAAGCT-3′/5′-GGGTCAGCACAGACCTCTCTCT-3′; *Ccl5* primer: 5′-CTGCTTGCTCTAGTCCA-3′/5′-ATGCTGATTTCTTGGGTTT-3′; *Gbp2* primer: 5′-GATGAACAAGCTAGCTGGGAAGAG-3′/5′-CCTTGGTGTGAGACTGCACAGT-3′. Data were calculated relative to the housekeeping gene *Actb* by using the 2^−ΔΔ^*^C^*^t^ method.

### Enzyme-linked immunosorbent assay

A flexible assay plate (BD Biosciences) was coated with anti-Dkk3-4.22 antibody (1 μg/ml). Tissues were homogenized in ProteoJET mammalian cell lysis reagent (Fermentas) supplemented with Complete, EDTA-free, protease inhibitor cocktail (Roche). Biotinylated goat anti-mouse Dkk3 antibody (R&D Systems, Minneapolis, MN, USA) at a final concentration of 1 μg/ml was used as detection antibody. Streptavidin-peroxidase (Jackson ImmunoResearch) diluted in PBS-Tween was added for 30 min at room temperature. After washing, orthophenylene diamine substrate at a concentration of 1 mg/ml in 100 mM sodium hydrogen phosphate buffer containing 0.03% peroxide was applied. The reaction was stopped with 2 M sulfuric acid. Optical densities were analyzed with a Viktor 2 photometer (Perkin Elmer) at 490 nm.

### Dorsal root ganglia

L2–L6 dorsal route ganglia (DRG) neurons isolation and cultured was performed as described (23). DRGs were excised and roots and membranes were removed and placed in sterile calcium- and magnesium-free PBS. Ganglia were collected and resuspended in DMEM/F12 (1:1) containing 10% horse serum, 2% penicillin–streptomycin solution, 1% l-glutamine, and 0.8% d-glucose (w/v). 0.1% collagenase was added and the tissue incubated at 37°C for 45 min. The tissue was incubated for a further 45 min in growth medium containing 0.05% trypsin at 37°C. Cells were dissociated by trituration through a fire-polished Pasteur pipette. After centrifugation at 250 × *g* for 5 min, the resultant pellet was washed twice in growth medium. Finally, cells were plated immediately, either onto a six-well plate or glass cover-slips which had previously been coated with poly l-ornithine (1 μg/ml) and laminin (25 μg/ml) supplemented with murine nerve growth factor (100 ng/ml). After 24 h incubation, the culture medium was supplemented with cytosine arabinoside (10 μM) and incubated for 12 h, after which time, culture medium was changed every 2 days until 70–80% confluence was reached.

### IFNγ injection into the hypothalamic arc

Bilateral stereotaxic injections were performed as described (25). Hundred nanograms of recombinant murine IFNγ (Peprotech) was injected into the hippocampus of each hemisphere (two injections per hemisphere: (1) caudal to bregma: −2.2 mm, lateral: −2.5 mm, ventral: 2.4 mm; (2) caudal to bregma: −2.0 mm, lateral: −2.0 mm, ventral: 1.6 mm). Twenty-four hours after injection, hippocampi of both hemispheres were isolated and used for analysis.

### Statistical analysis

All data are represented as mean ± SEM. Statistical analysis was performed using the GraphPad Prism 6 software. *p* values were calculated using an unpaired two-tailed Student’s *t*-test. *p* < 0.05 was considered significant.

## Results

### Dkk3 deficiency does not influence T-cell development, activation, and memory formation in the steady state

**Figure 1:**
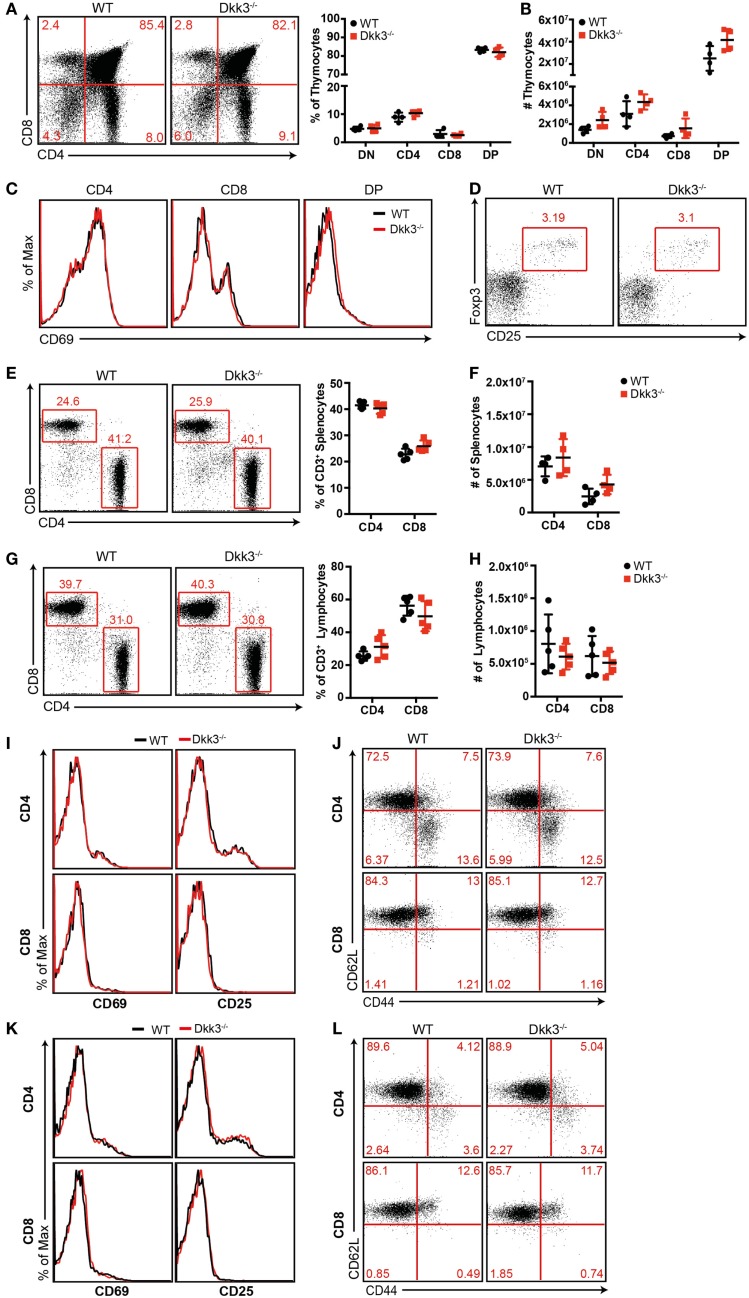
**Dkk3 does not influence T-cell development and activation in naïve Dkk3-deficient mice**. **(A)** Thymocytes from naïve Dkk3^−/−^ mice and littermate controls were stained for CD4 and CD8 and analyzed by flow cytometry. Distribution of thymocyte subsets (DN: CD4^−^ CD8^−^ double negative; DP: CD4^+^ CD8^+^ double positive) is shown in a representative dot plot (left) and cumulative data of percentages of whole thymocytes (right; *n* = 4–5). **(B)** Absolute numbers of respective thymocyte subsets from **(A)**, determined by flow cytometry (*n* = 4–5). **(C)** Flow cytometric analysis of CD69 cell surface expression on CD4^+^, CD8^+^, and CD4^+^ CD8^+^ double positive (DP) subsets from **(A)**. One representative histogram is shown (*n* = 4–5). **(D)** Representative dot plot of CD25^+^ Foxp3^+^ cells among CD4 single positive cells in the thymus of Dkk3^−/−^ and WT control mice. **(E)** Analysis of CD4^+^ and CD8^+^ T-cell subsets, among CD3 positive cells, in the spleen of Dkk3^−/−^ mice compared to littermate controls. One representative dot plot (left) as well as respective cumulative data is shown. **(F)** Absolute numbers of lymphocyte subsets from **(D)**, determined by flow cytometry (*n* = 4–5). **(G)** Analysis of CD4^+^ and CD8^+^ T-cell subsets among CD3 positive cells in peripheral lymph nodes (LN) of Dkk3^−/−^ mice and WT controls. One representative dot plot (left) as well as respective cumulative data is shown. **(H)** Absolute numbers of lymphocyte subsets from **(F)**, determined by flow cytometry (*n* = 4–5). **(I+K)** Flow cytometric analysis of surface expression of the activation markers CD25 and CD69 on CD4^+^ (upper row) and CD8^+^ (lower row) T cells in **(I)** spleen and **(K)** peripheral lymph nodes of naïve Dkk3^−/−^ (red) and WT control (black) mice. Shown is one representative histogram (*n* = 10). **(J+L)** Flow cytometric analysis of the surface expression of CD44 and CD62L on CD4^+^ (upper row) and CD8^+^ (lower row) T cells in **(J)** spleen and **(L)** peripheral lymph nodes of naïve Dkk3^−/−^ and WT control mice. Shown is one representative dot plot (*n* = 8).

In order to identify the impact of Dkk3 on T cells of a polyclonal repertoire, we initially analyzed T-cell development in Dkk3-deficient mice. Flow cytometric analysis revealed similar percentages (Figure 1A) and numbers (Figure 1B) of thymocyte subsets in Dkk3^−/−^ and WT control mice. Additionally, cell surface expression of CD69, which is a marker for the engagement of the TCR-mediated positive selection process in the thymus, was identical on the different thymocyte subsets in both strains (Figure 1C). Frequencies of CD4^+^ CD25^+^ Foxp3^+^ regulatory T cells in the thymus were not altered in Dkk3^−/−^ mice (Figure 1D). The ratio and numbers of CD4^+^ and CD8^+^ T cells in spleen (Figures 1E,F) and peripheral lymph nodes (Figures 1G,H) were also comparable. Moreover, analysis of TCR-Vβ expression showed that genetic Dkk3 deletion did not strongly affect the development of the TCR repertoire (Table 1). In addition to their developmental properties, we analyzed the activation and memory status of T cells in naïve Dkk3^−/−^ mice. Surface expression of the activation markers CD25 and CD69 on CD4^+^ as well as CD8^+^ T cells from spleen (Figure 1I) and lymph nodes (Figure 1K) were comparable in Dkk3^−/−^ and WT control mice. Similarly, Dkk3 deficiency did not influence the expression of the memory markers CD44 and CD62L on CD4^+^ and CD8^+^ T cells in spleen (Figure 1J) and lymph nodes (Figure 1L). Thus, these results illustrate that Dkk3 does not interfere with T-cell development, activation, and memory differentiation in the steady state.

**Table 1:** **Normal T-cell repertoire in Dkk3-deficient mice**.

**THYMUS CD4**														
**TCR-Vβ**	**2**	**3**	**4**	**5.1/5.2**	**6**	**7**	**8.1/8.2**	**9**	**10b**	**11**	**12**	**13**	**14**	**17a**
**WT**	0.5	3.9	2.4	2.8	6.2	1.3	8.6	8.0	3.7	8.0	1.2	1.3	3.8	3.0
**Dkk3^−/−^**	0.5	4.5	4.3	2.8	4.6	1.1	10.6	6.8	5.1	6.8	0.7	1.0	3.3	3.3

**THYMUS CD8**														
**TCR-Vβ**	**2**	**3**	**4**	**5.1/5.2**	**6**	**7**	**8.1/8.2**	**9**	**10b**	**11**	**12**	**13**	**14**	**17a**
**WT**	0.1	4.3	1.4	5.8	11.5	1.7	8.4	5.8	1.0	0.9	0.5	0.5	2.0	2.1
**Dkk3^−/−^**	0.1	5.5	3.3	4.6	9.5	1.6	9.5	6.1	1.6	1.2	0.2	0.2	2.5	1.6

**SPLEEN CD4**														
**TCR-Vβ**	**2**	**3**	**4**	**5.1/5.2**	**6**	**7**	**8.1/8.2**	**9**	**10b**	**11**	**12**	**13**	**14**	**17a**
**WT**	0.6	4.9	2.6	2.3	3.1	1.0	7.5	8.7	0.6	3.5	0.7	1.0	3.6	2.5
**Dkk3^−/−^**	0.7	3.6	1.8	2.2	3.4	1.7	6.9	6.3	0.5	3.3	2.9	1.0	2.2	2.3

**SPLEEN CD8**														
**TCR-Vβ**	**2**	**3**	**4**	**5.1/5.2**	**6**	**7**	**8.1/8.2**	**9**	**10b**	**11**	**12**	**13**	**14**	**17a**
**WT**	0.2	5.7	1.6	4.7	11.7	0.9	6.2	6.7	0.2	0.6	0.5	0.3	1.9	0.9
**Dkk3^−/−^**	0.3	4.2	0.7	5.2	8.0	0.6	5.5	4.3	1.1	0.9	1.5	0.6	1.1	0.5

*Flow cytometric analysis of TCR-V β chain expression on T cells. Percentages of CD4 ^+^ or CD8 ^+^ T cells in thymus and spleen carrying the respective TCR-V β*.

### Dkk3 contributes to the acceptance of embryoid body grafts

**Figure 2:**
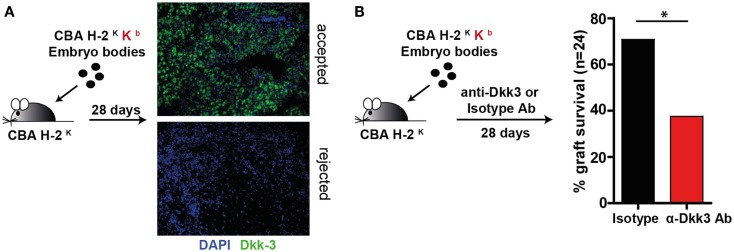
**Dkk3 protects MHC class-I mismatched transplanted embryoid bodies from rejection**. **(A)** Male CBK mice-derived embryoid bodies were transplanted under the kidney capsule of male CBA mice. Twenty-eight days after transplantation, remaining embryoid body tissue was stained with anti-Dkk3 antibody. Shown is one representative staining of accepted and remaining elements of the rejected tissue (*n* = 3). **(B)** Male CBK mice-derived embryoid bodies were transplanted under the kidney capsule of male CBA mice. Seven days later, 1 mg anti-Dkk3 or isotype control antibody was administered, followed by 0.5 mg every fourth day. Twenty-eight days after transplantation, the acceptance rate of the transplants was determined (Log-rank test, *n* = 24, *p < 0.05).

Next, we were interested in the influence of Dkk3 on T-cell function under stimulatory conditions. As we previously identified Dkk3 to be crucial for the establishment of peripheral CD8 T-cell tolerance in a TCR transgenic system (20), we wondered whether it may also influence CD8 T-cell responses in a polyclonal TCR repertoire. To address this question, we employed an EBs transplantation model. EBs are embryonic stem cell-derived composite tissues that have been previously reported to establish an immunosuppressive micromilieu (22). In previous work, it has been demonstrated that transplantation of male MHC class-I mismatched EBs under the kidney capsule of sex-matched recipient mice results in 50% graft acceptance (22). Here, male CBK-derived EBs were transplanted under the kidney capsule of male CBA recipient mice. The accepted and rejected transplants were analyzed at day 28 for Dkk3 expression. Strikingly, Dkk3 protein was detected in the accepted but not in the remains of the rejected grafts (Figure 2A). To dissect the contribution of Dkk3 to the establishment of the local immunosuppressive microenvironment of the EB grafts, we administered anti-Dkk3 neutralizing or isotype control antibody following transplantation and assessed its effect on graft survival. Interestingly, blocking of Dkk3 by the use of the monoclonal antibody resulted in an increased incidence graft rejection (Figure 2B). Taken together, our results demonstrate that Dkk3 expression correlates with the development of a local immunosuppressive micromilieu and the immunosuppressive function of Dkk3 contributes to the acceptance of MHC class-I mismatched EB grafts.

### Dkk3^−/−^ mice suffer from exacerbated experimental autoimmune encephalomyelitis

**Figure 3:**
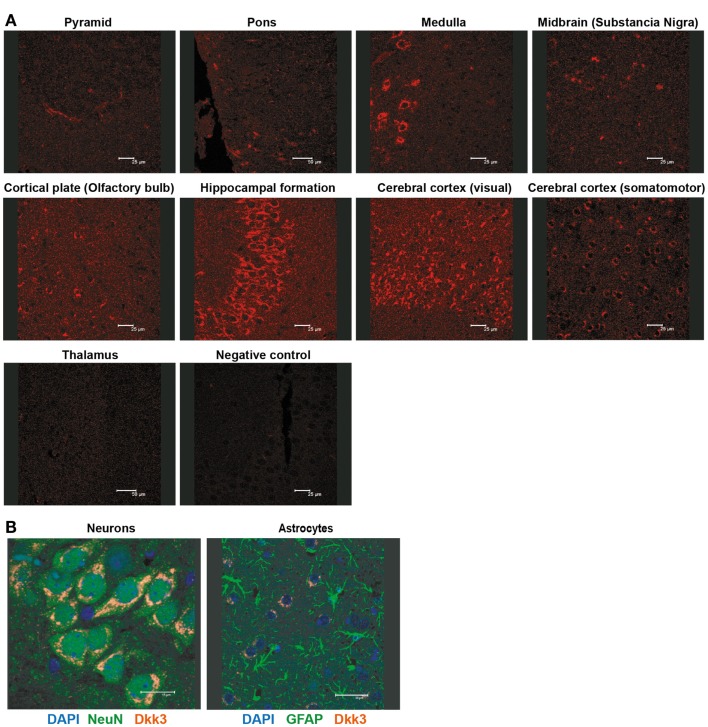
**Dkk3 is expressed by neurons in distinct areas of the brain**. **(A)** Brain sections of C57BL6 mice were stained with anti-Dkk3 antibody. Brain regions were identified by morphology and location (Scale bar: 25/50 μm). **(B)** Respective brain sections from **(A)** were stained with anti-Dkk3 (red) and either anti-neuronal nuclei (NeuN, green, left panel) or anti-Glial fibrillary acidic protein (GFAP, green, right panel) antibodies. Nuclei were counterstained with DAPI (Scale bar: 15/30 μm).

Dkk3 was reported to be strongly expressed in the brain (15). Detailed immune-histological analysis revealed a highly specific expression pattern within the organ. Prominent Dkk3 production was restricted to the hippocampus, medulla, and the visual area of the cerebral cortex (Figure 3A). Moreover, double-fluorescence staining indicated that neurons (NeuN^+^) but not astrocytes (GFAP^+^) are the main source of Dkk3 in the brain (Figure 3B).

**Figure 4:**
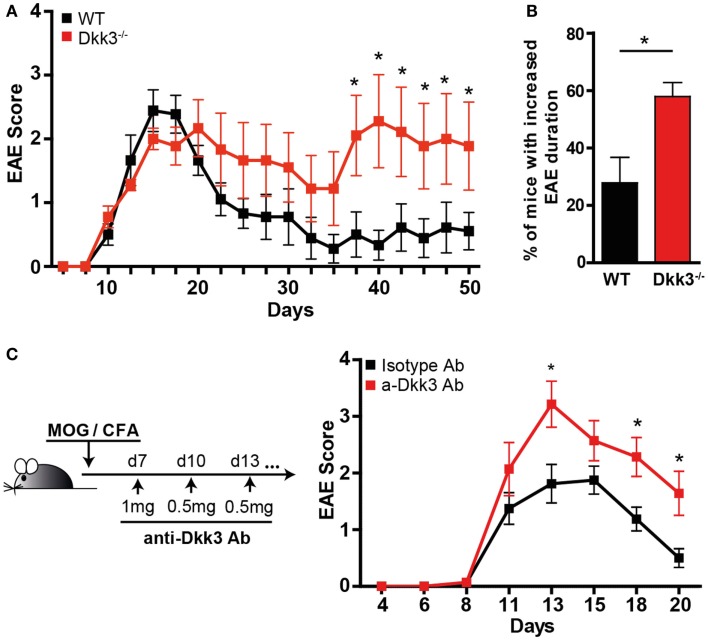
**Dkk3-deficient mice suffer from prolonged and exacerbated EAE**. **(A)** Dkk3^−/−^ and WT mice were immunized s.c. with MOG_33–55_ peptide in CFA. The mean EAE scores of one representative out of three experiments are shown (*n* = 9). **(B)** The percentage of living mice with disease duration longer than 20 days was determined. Results from two independent experiments were pooled (*n* = 16, *p < 0.05). **(C)** C57BL6 mice received either anti-Dkk3 or isotype control antibody at days 7 (1 mg/mouse), 10, 13, 16, 21 (0.5 mg/mouse) after MOG_33–55_/CFA immunization. Shown is one representative experiment out of three (*n* = 10, *p < 0.05).

Based on our findings that Dkk3 in the microenvironment might influence CD8 T-cell responses (Figure 2), we wondered whether it acts on local CD4 T-cell reactivity as well. Due to the locally high expression of Dkk3 in the brain, we chose the EAE as a model system to answer this question. EAE is regarded as a mouse-model of human multiple sclerosis, in which auto-reactive CD4 T-cell-mediated demyelization is triggered in the CNS. Thus, EAE was induced by immunization with a MOG_33–55_ peptide and CFA in Dkk3^−/−^ and WT mice and the disease course was followed. The onset of EAE and the disease incidence were comparable in the two mouse groups (Figure 4A; Table 2). Moreover, the severity of EAE in the acute phase was not significantly higher after genetic deletion of Dkk3 (Figure 4A; Table 2). However, Dkk3^−/−^ mice failed to recover and exhibited a more chronic disease when compared to WT controls (Figure 4A). Most of the EAE-affected WT mice recovered after initial paralysis and only 27.9% had clinical symptoms for longer than 20 days. In contrast, the majority of diseased Dkk3^−/−^ mice exhibited increased disease duration, developing chronic disease or suffering from relapses, as 57.9% had clinical symptoms for longer than 20 days (Figure 4B).

**Table 2:** **Onset and severity in the acute phase of experimental autoimmune encephalomyelitis in Dkk3-deficient mice was comparable to WT controls**.

	Incidence	%	Day of disease onset	Mean maximal disease severity	Dead mice
WT	24/26	92.3	11.1 ± 2.6	2.4 ± 1.1	1
Dkk3^−/−^	25/25	100	10.8 ± 2.3	3.1 ± 1.1	4

We next sought to assess whether an established autoimmune process is also susceptible to the effects of Dkk3. For this, we treated mice 1 week after EAE induction with the neutralizing anti-Dkk3 antibody. Due to changes in the blood–brain barrier during EAE, antibodies can access inflammatory lesions in the CNS (24). We found that Dkk3 blockade starting 1 week after disease induction resulted in a significant increase and prolongation of EAE symptoms, as compared to isotype-treated controls (Figure 4C) suggesting that Dkk3 controls the potency and duration of the T-cell effector phase, locally in the CNS.

To explore the consequences of Dkk3 deficiency on T-cell function in EAE more detailed, we analyzed the T-cell phenotype in the CNS of diseased Dkk3^−/−^ and WT control mice. While IL-17- and GM-CSF-producing CD4^+^ T cells, have been shown to play an important role in EAE induction (25, 26), IFNγ was reported to have a pleiotropic effect in EAE. Depending on the cell type of origin and time point of expression, it may mediate disease-promoting or disease-limiting effects (27). Therefore, we re-stimulated lymphocytes isolated from the CNS with MOG peptide and assessed the numbers of IL-17, GM-CSF, and IFNγ-producing CD4 T cells. Since the MOG_33–55_ peptide also contains a CD8-epitope able to induce IFNγ production and EAE (28), we also analyzed CD8^+^ IFNγ^+^ T cells. The presence of CD4^+^ CD25^+^ Foxp3^+^ regulatory T cells, which are shown to be potent down-modulators of the T-cell response in EAE (29), was also determined.

**Figure 5:**
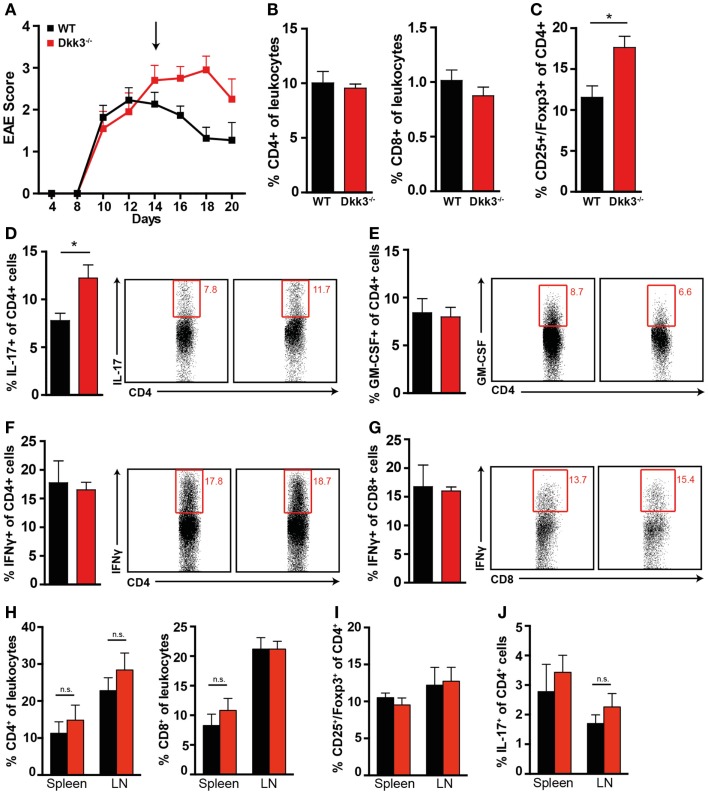
**Increased proportions of CD4^+^ CD25^+^ Foxp3^+^ regulatory T cells and IL-17-producing CD4^+^ T cells in the CNS but not in the draining lymph nodes (Figure 6H) of Dkk3^−/−^ at the peak of EAE**. **(A)** EAE was induced in Dkk3^−/−^ and WT mice by immunization with MOG_33–55_ in CFA. Mean clinical EAE score over time is displayed. At the indicated time point (black arrow, 14 days after EAE induction), half of the mice were sacrificed and lymphocytes were isolated from brain and spinal cord for analysis in **(B–G)** (*n* = 8). **(B)** Percentages of CD4^+^ and CD8^+^ T cells among leukocytes in the CNS of Dkk3^−/−^ and WT mice. **(C)** Proportion of CD25^+^ Foxp3^+^ of CD4^+^ cells (*p < 0.05). **(D–G)** Isolated lymphocytes from **(A)** were re-stimulated *in vitro* with 50 μg/ml MOG_33–55_ peptide for 6 h in the presence of Golgi Plug and intracellularly stained for the respective cytokines. Shown is one representative dot plot (left panel) and cumulative data of three individual experiments (*n* = 8). **(D)** Percentages of IL-17^+^ of CD4^+^ T cells (*p < 0.05). **(E)** Percentages of GM-CSF^+^ of CD4^+^ T cells. **(F)** Percentages of IFNγ^+^ of CD4^+^ T cells. **(G)** Percentages of IFNγ^+^ of CD8^+^ T cells. **(H)** Percentages of CD4^+^ and CD8^+^ T cells in spleen and peripheral lymph nodes (LN) of respective mice from **(A)**. **(I)** Percentages of CD25^+^ Foxp3^+^ of CD4^+^ cells in spleen and draining lymph nodes (LN) of respective mice from **(A)**. **(J)** Percentages of IL-17^+^ of CD4^+^ cells in spleen and draining lymph nodes (LN) of respective mice from **(A)**.

At the peak of the disease, when EAE symptoms in Dkk3^−/−^ and WT mice were still comparable (Figure 5A), the numbers of infiltrating CD4^+^ and CD8^+^ T cells in the CNS were similar (Figure 5B). However, while GM-CSF and IFNγ producing cells were not affected (Figures 5E–G), the proportion of IL-17-producing CD4^+^ T cells was slightly increased in the CNS of Dkk3-deficient mice (Figure 5D). Interestingly, at the same time, the proportion of CD4^+^ CD25^+^ Foxp3^+^ regulatory T cells was significantly elevated (Figure 5C). These differences were absent in spleen and draining lymph nodes (Figures 5H–J) indicating a local effect of Dkk3.

**Figure 6:**
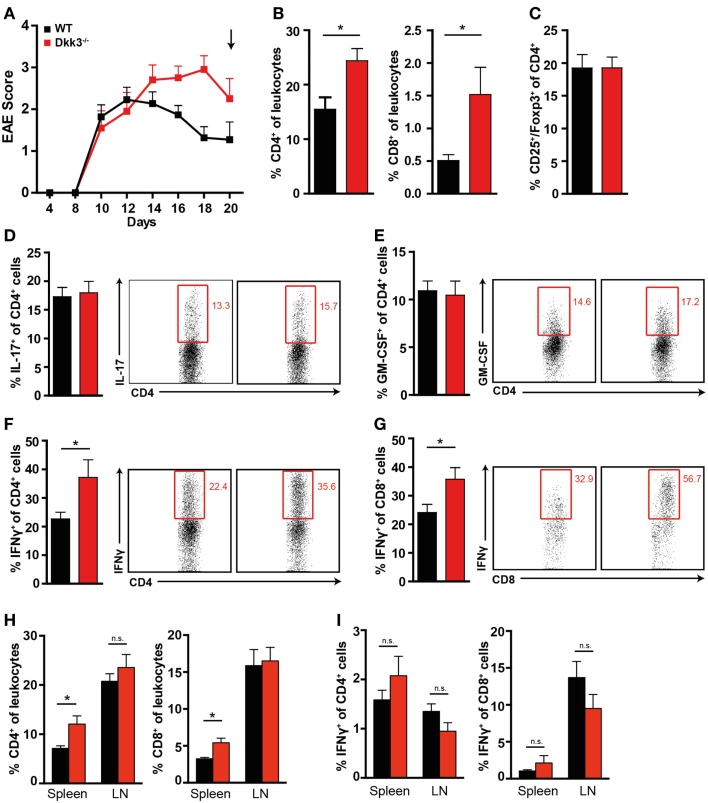
**Increased numbers of IFNγ-producing T cells in the CNS of EAE diseased Dkk3^−/−^ mice in the recovery phase**. **(A)** EAE was induced in Dkk3^−/−^ and WT mice by immunization with MOG_33–55_ in CFA. Mean clinical EAE score over time is displayed. At the indicated time point (black arrow, 20 days after EAE induction), mice were sacrificed and lymphocytes were isolated from brain and spinal cord for analysis in **(B–G)**. **(B)** Percentages of CD4^+^ and CD8^+^ T cells among leukocytes in the CNS of Dkk3^−/−^ and WT mice (*p < 0.05). **(C)** Proportion of CD25^+^ Foxp3^+^ of CD4^+^ cells. **(D–G)** Isolated lymphocytes from **(A)** were re-stimulated *in vitro* with 50 μg/ml MOG_33–55_ peptide for 6 h in the presence of Golgi Plug and intracellularly stained for the respective cytokines. Shown is one representative dot plot (left panel) and cumulative data of three individual experiments (*n* = 8). **(D)** Percentages of IL-17^+^ of CD4^+^ T cells. **(E)** Percentages of GM-CSF^+^ of CD4^+^ T cells. **(F)** Percentages of IFNγ^+^ of CD4^+^ T cells (*p < 0.05). **(G)** Percentages of IFNγ^+^ of CD8^+^ T cells (*p < 0.05). **(H)** Percentages of CD4^+^ and CD8^+^ T cells in spleen and draining lymph nodes (LN) of respective mice from **(A)**. **(I)** Percentages of IFNγ^+^ of CD4^+^ and CD8^+^ cells in spleen and draining lymph nodes (LN) of respective mice from **(A)**.

In contrast, during the recovery phase of the disease, when more severe EAE symptoms in Dkk3^−/−^ mice were manifest (Figure 6A), we found higher numbers of CD4^+^ and CD8^+^ T cells in the CNS but not in the draining lymph nodes (Figure 6H) of Dkk3-deficient mice compared to WT controls (Figure 6B). Furthermore, the ratio of IFNγ-producing CD4^+^ and CD8^+^ was significantly increased (Figures 6F,G) while the proportion of IL-17 and GM-CSF-producing CD4^+^ T cells (Figures 6D,E) as well as CD4^+^ CD25^+^ Foxp3^+^ cells (Figure 6C) was similar in the CNS of both strains. The impact on the T-cell phenotype was again a brain-specific effect, since Dkk3 deficiency did not interfere with the levels of IFNγ-producing CD4^+^ and CD8^+^ T cells in spleen and draining lymph nodes (Figure 6I). Thus, our data indicate that Dkk3 modulates T-cell activation and polarization during the advanced phase of EAE locally in the CNS and consequently promotes symptom recovery.

### Dkk3 serves as part of an IFNγ negative feedback loop in the brain during EAE

**Figure 7:**
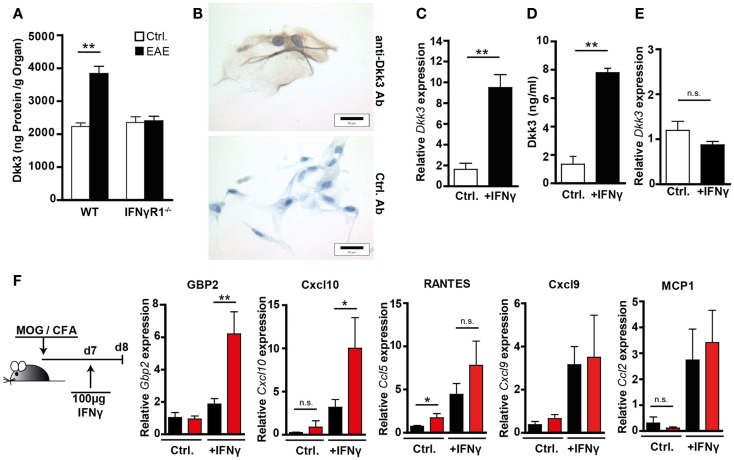
**Neuronal Dkk3 expression is induced by IFNγ and limits IFNγ induced gene expression in the brain**. **(A)** Dkk3 protein levels in brain lysates of healthy and EAE-diseased WT and IFNγR1^−/−^ mice were measured by ELISA. EAE was induced by immunization with MOG/CFA and minimal clinical EAE score of used mice was 4. Protein amount was related to organ weight (*n* = 5, **p < 0.01). **(B)** Histological analysis of *ex vivo* cultured, primary dorsal route ganglia (DRG) stained with anti-Dkk3 (upper row) or isotype control antibody (lower row). **(C)** QRT-PCR data showing relative *Dkk3* expression in cells from **(C)** (*n* = 3, **p < 0.01). **(D)** Dkk3 ELISA of supernatant from control and IFNγ stimulated primary DRG isolated from WT mice. DRG were stimulated for 24 h with 100 ng/ml IFNγ (*n* = 3, **p < 0.01). **(E)** QRT-PCR data showing relative Dkk3 expression in control and IFNγ stimulated primary DRG from IFNγR1^−/−^ mice. DRG were stimulated for 24 h with 100 ng/ml IFNγ (*n* = 3). **(F)** WT and Dkk3^−/−^ were immunized with MOG_33–55_ in CFA. Seven days later, 100 ng recombinant IFNγ in PBS was injected stereotactically into hippocampi of the respective mice. One day later, hippocampi were harvested and RNA was isolated. As controls, hippocampi of non-IFNγ-injected mice, 8 days after MOG/CFA immunization were used. Shown are qRT-PCR data of the relative expression of *Gbp2*, *Cxcl10*, *Cxcl9*, *Ccl2* (MCP-1), *Ccl5* (RANTES) in respective hippocampi (*n* = 8, *p < 0.05, **p < 0.01).

IFNγ plays a multifaceted role in EAE. Its disease-limiting effect was suggested to be partially mediated by the induction of anti-inflammatory mediators like IDO (14) or nitric oxide (NO) (30). Despite elevated IFNγ levels in the CNS, we observed a prolonged disease process in the Dkk3-deficient situation. Therefore, we hypothesized that Dkk3 might be part of an IFNγ-mediated regulatory mechanism, that is lost in Dkk3^−/−^ mice. In order to test this, we initially analyzed the capacity of IFNγ to induce Dkk3 expression during EAE in the brain. Comparison of Dkk3 expression in the brain of healthy controls and EAE-bearing mice, at the peak of diseases, revealed a significant increase of Dkk3 levels in the inflamed brains of WT mice. However, such an elevation was not detectable in brains of IFNγR1^−/−^ mice (Figure 7A), indicating an IFNγ-dependent induction of Dkk3 expression in EAE brains. In order to find out whether IFNγ is able to up-regulate Dkk3 expression in neuronal cells, we made use of *ex vivo* cultured primary DRG neurons. Histological analysis revealed, just as for neurons *in vivo*, that these cells express Dkk3 (Figure 7B). Moreover, IFNγ stimulation of DRGs increased the basal expression level (Figure 7C) and secretion of Dkk3 protein (Figure 7D) by nearly sixfold. As expected, this up-regulation was not observed in IFNγR1^−/−^ control DRGs (Figure 7E). Thus, IFNγ up-regulates Dkk3 expression in neuronal cells.

In order to find out whether Dkk3 is, in turn, capable of influencing IFNγ activity during EAE, we analyzed IFNγ-induced gene expression in the brain. To enhance the pathological situation, in which the first IFNγ-producing T cells enter the brain 7 days after EAE induction (31), we performed the following experiment: 7 days after EAE induction in Dkk3^−/−^ and WT mice, IFNγ was stereotactically injected into the hippocampal arc of the mice, and expression of IFNγ target genes was analyzed 24 h after administration. This area was targeted because of the high local concentration of Dkk3 in the hippocampus (Figure 3A). As a control, hippocampi of non-injected animals were analyzed. QRT-PCR analysis revealed that IFNγ injection strongly induced IFNγ target gene expression in hippocampi of injected mice compared to non-injected controls. Furthermore, this induction was more prominent in Dkk3-deficient mice. In Dkk3^−/−^ hippocampi, the expression of Gbp2 and Cxcl10 was significantly enhanced upon IFNγ administration. However, Cxcl9, Ccl2, and Ccl5 expression was not much altered (Figure 7F). In control hippocampi, dkk3 deficiency did not cause an increase in IFNγ target gene expression (Figure 7F). In summary, these data suggest that Dkk3 negatively modulates IFNγ activity in the EAE brain.

## Discussion

Regulation of T-cell responses within tissues is essential for the preservation of organ integrity and function. In this context, the tissue microenvironment plays a pivotal role in the establishment of an immunological balance. In this study, we provide evidence that the secreted glycoprotein Dkk3 functions as a tissue-derived modulator of local T-cell responses. Our data show that Dkk3 (i) is part of the local microenvironment that protects transplanted MHC class-I mismatched embryo bodies from CD8^+^ T-cell-dependent rejection and (ii) contributes to the control of CD4^+^ T-cell-mediated autoimmune encephalomyelitis by limitation of T-cell activation and polarization as well as IFNγ activity in the CNS.

Our group previously demonstrated that the presence of Dkk3 is essential for the establishment of peripheral CD8^+^ T-cell tolerance in a TCR/MHC class-I double transgenic mouse system. Genetic deletion or antibody blockade of Dkk3 in this system led to an increased local CD8^+^ T-cell reactivity resulting in an enhanced, antigen-specific eradication of tumors and skin grafts (20). The investigations performed here display a similar, immune-modulatory role of Dkk3 for the polyclonal T-cell repertoire *in vivo*. Transplanted EBs were shown to create an immune suppressive microenvironment by, for example, the release of transforming growth factor-β (TGF-β) (22). EB transplants were shown to express Dkk3 and antibody-mediated blockade of Dkk3 increased the frequency of their rejection, albeit some EB grafts remaining intact. We therefore, suggest that Dkk3 represents one constituent of the local, suppressive EB micromilieu suppressing cytotoxic CD8^+^ T-cell responses. This view is supported by the observation that the global absence of Dkk3 did not lead to a systemic hyperresponsiveness of cytotoxic CD8^+^ T cells *in vivo* in the polyclonal repertoire (20).

In contrast to the double transgenic system in which Dkk3 was expressed by a unique, tolerant CD8^+^ T-cell population, (20), Dkk3 was reported to be mainly produced by tissue cells but very rarely in the hematopoietic compartment in the physiological situation. Dkk3 expression in the hematopoietic system was only detected at a transcriptional level in a CD27 negative, IL-17-producing subset of γδ T cells (32) and long-term memory CD8 T cells (33) but not in naïve or activated conventional αβ T cells. Highest Dkk3 levels were found in organs like uterus, placenta, eye, and brain (15, 17). These organs are classically considered to be immune-privileged, a situation in which the tissue is protected from otherwise damaging immune responses. This is of particular importance as their regenerative capacity is very low and cellular damage would strongly impair organ integrity and function (34, 35). Based on the immune suppressive capacity of Dkk3 shown here, one could speculate that Dkk3 is one contributor to the creation of such immunosuppressive microenvironments.

Indeed, our studies on EAE demonstrate an important role of Dkk3 in the regulation of CD4^+^ T-cell responses. Dkk3 deficiency resulted in a delayed EAE remission with exacerbated symptoms during recovery phase but did not lead to higher EAE susceptibility, premature disease onset nor increased severity at the peak of disease. These data indicate that Dkk3 mediates its function rather locally during the T-cell effector phase, rather than systemically in spleen and lymph nodes during the T-cell priming phase. This assumption is supported by the finding that blockade of Dkk3 by a monoclonal antibody, starting 7 days after immunization, is sufficient to induce a similar phenotype. The locally restricted function might also explain, at least in part, why Dkk3 deficiency does not lead to T-cell activation in secondary lymphoid organs in the steady state and thus, why Dkk3^−/−^ mice do not suffer from spontaneous autoimmunity (21).

The observed shift of T-cell polarization within the CNS of EAE-bearing Dkk3-deficient mice varied at different stages of the disease. At the peak of disease, a higher proportion of Th17 and Foxp3^+^ regulatory T cells was detected. While Th17 cells are crucial for the induction of EAE (25, 26), Foxp3^+^ Tregs were shown to suppress encephalitogenic T-cell reactivity (29). As both subsets were enriched in the Dkk3^−/−^ CNS, it could be that they compensate each others function, resulting in a similar EAE score compared to WT mice. However, in the recovery phase, the heightened EAE severity in Dkk3-deficient mice was accompanied by elevated T-cell numbers and an increase in IFNγ producing CD4^+^ and CD8^+^ T cells in the CNS. Again, the altered T-cell polarization in the CNS of Dkk3-deficient mice upon EAE induction was restricted to the brain and not observed in the secondary lymphoid organs endorsing the hypothesis of local Dkk3 action.

IFNγ has a multifaceted role in EAE. On the one hand, it has been positively correlated with disease severity (36), while on the other hand genetic or antibody-mediated neutralization exhibited disease-ameliorating effects (37–39). It was suggested that, after being pro-inflammatory and disease-promoting in the early phase, IFNγ exerts an anti-inflammatory, beneficial function in the later phases (27). Its disease-providing properties are mediated by induction of antigen presentation, promotion of Th1 differentiation as well as up-regulation of adhesion molecules and the induction of a wide range of pro-inflammatory mediators (40). The anti-inflammatory function is attributed to the inhibition of T-cell expansion, induction of Foxp3 positive regulatory cells, and the up-regulation of anti-inflammatory mediators like IDO or the inducible nitric oxide synthase (iNOS) (27, 30, 41). Our studies reveal that Dkk3 expression is up-regulated in the brain of EAE-bearing mice in an IFNγ-dependent manner. Moreover, we found that Dkk3 is able to regulate the expression of certain IFNγ target genes in the brain. Together these results suggest that neuron-derived Dkk3 might serve as a mediator of the anti-inflammatory arm of IFNγ during EAE, conducting its function by regulation of IFNγ activity in a negative feedback mechanism.

The immune modulator IDO also locally suppresses T-cell responses by the catabolic deprivation of tryptophan and production of kynurenine metabolites, and is thus, thought to play an important role in the induction of immune tolerance and the prevention of autoimmunity (42, 43). It is expressed in the brain and is further up-regulated by IFNγ. Chemical inhibition or genetic deletion of IDO causes exacerbated EAE symptoms (14, 44). Moreover, IDO deficiency leads to increased numbers of IFNγ-producing T cells in EAE-bearing mice and treatment of transferred EAE with kynurenines shifts the cellular immune response from Th1 to Th2 (45). These results led to the assumption that IDO expression in the CNS initiates a negative feedback loop to self-limit autoimmunity (44). In addition, via the interaction with its ligand PD-1, tissue-derived PD-L1 is important for the establishment of peripheral tolerance. In a fully MHC mismatched cardiac allograft model, blockade of PD-L1 abrogated CTLA4Ig-induced tolerance and accelerated organ rejection. This was associated with an increase in the frequency of IFNγ-producing allo-reactive T cells and expansion of CD8 effector cells (46). High PD-L1expression was shown to be key for maintaining immune privilege in the eye (47) and feto-maternal tolerance in the placenta (48). PD-L1-deficient mice do not display T-cell activation *per se* or spontaneous autoimmunity in the steady state. However, upon MOG immunization, these mice suffer from exacerbated EAE (49). The functional characteristics of Dkk3 identified here seem quite similar to those of these well-characterized immune-modulatory molecules.

Mechanistically, Dkk3 does not conduct its function via interference with the development of the TCR repertoire. This is indicated by the findings that the TCR-Vβ frequencies are not dissimilar, that antibody blockade reflects the phenotype of genetic deletion, and that immune responses in the lymphoid compartments are not influenced by Dkk3 (20). Our finding that Dkk3 interferes with the expression of IFNγ target genes like Gbp2 or Cxcl10 suggests that Dkk3 may not necessarily act directly on T cells but may also affect the function of ambient cells in the microenvironment which in turn modulate T-cell responses. For example, oligodendrocytes and astrocytes were shown to up-regulate the expression of Gbp2 and Cxcl10 in response to IFNγ during EAE (50, 51). Since Dkk3 may act as Wnt pathway modulator (17–19), it is possible that its immune-modulatory function also relies on this property, especially as Wnt signaling is known to influence T-cell effector function (52) and linage commitment (53–55). However, the distinct cellular and molecular mechanisms through which Dkk3 mediates its function warrant further investigation.

In summary, our results strongly suggest that Dkk3 contributes to the establishment of peripheral T-cell tolerance and the protection of tissues from self-destructive immune responses. These findings suggest that Dkk3 should be considered as a potential target for therapeutic approaches in transplant rejection and autoimmunity.

## Author Contributions

MM and MP contributed equally to this work. MM, MP, VN, VK, JL, KL, and AB performed experiments and analyzed data. MM, MP, TO, and BA designed experiments with contributions of TF, GM, PN, HG, and HW. MM, MP, and BA wrote the manuscript.

## Conflict of Interest Statement

The authors declare that the research was conducted in the absence of any commercial or financial relationships that could be construed as a potential conflict of interest.
